# Predation efficiency of *Anopheles gambiae *larvae by aquatic predators in western Kenya highlands

**DOI:** 10.1186/1756-3305-4-128

**Published:** 2011-07-05

**Authors:** Eliningaya J Kweka, Guofa Zhou, Thomas M Gilbreath, Yaw Afrane, Mramba Nyindo, Andrew K Githeko, Guiyun Yan

**Affiliations:** 1 Centre for Global Health Research, Kenya Medical Research Institute, P. O. Box 1578, Kisumu 40100, Kenya; 2 Kilimanjaro Christian Medical College, Tumaini University, P. O. Box 2240, Moshi, Tanzania; 3 Program in Public Health, University of California, Irvine, CA 92697, USA; 4 Ecology and Evolutionary Biology, University of California, Irvine, CA 92697, USA

## Abstract

**Background:**

The current status of insecticide resistance in mosquitoes and the effects of insecticides on non-target insect species have raised the need for alternative control methods for malaria vectors. Predation has been suggested as one of the important regulation mechanisms for malaria vectors in long-lasting aquatic habitats, but the predation efficiency of the potential predators is largely unknown in the highlands of western Kenya. In the current study, we examined the predation efficiency of five predators on *Anopheles gambiae *s.s larvae in 24 hour and semi- field evaluations.

**Methods:**

Predators were collected from natural habitats and starved for 12 hours prior to starting experiments. Preliminary experiments were conducted to ascertain the larval stage most predated by each predator species. When each larval instar was subjected to predation, third instar larvae were predated at the highest rate. Third instar larvae of *An. gambiae *were introduced into artificial habitats with and without refugia at various larval densities. The numbers of surviving larvae were counted after 24 hours in 24. In semi-field experiments, the larvae were counted daily until they were all either consumed or had developed to the pupal stage. Polymerase chain reaction was used to confirm the presence of *An. gambiae *DNA in predator guts.

**Results:**

Experiments found that habitat type (*P *< 0.0001) and predator species (*P *< 0.0001) had a significant impact on the predation rate in the 24 hour evaluations. In semi-field experiments, predator species (*P *< 0.0001) and habitat type (*P *< 0.0001) were significant factors in both the daily survival and the overall developmental time of larvae. Pupation rates took significantly longer in habitats with refugia. *An. gambiae *DNA was found in at least three out of ten midguts for all predator species. *Gambusia affins *was the most efficient, being three times more efficient than tadpoles.

**Conclusion:**

These experiments provide insight into the efficiency of specific natural predators against mosquito larvae. These naturally occurring predators may be useful in biocontrol strategies for aquatic stage *An. gambiae *mosquitoes. Further investigations should be done in complex natural habitats for these predators.

## Background

Mosquitoes of the *Anopheles gambiae *complex contain the most efficient vector species for malaria transmission in sub-Saharan Africa [1]. Controlling *An. gambiae *s.l. (hereafter referred to as *An. gambiae*) populations is a priority in reducing malaria incidence in both endemic and epidemic areas. In recent years, there has been evidence of increasing resistance of *An. gambiae *to pyrethroids and dichloro-diphenyl-trichloroethane (DDT) which are used in bed net treatment and indoor residual spraying respectively [2,3].

There are concerns about the effect of chemicals on non-target organisms, including beneficial and non-beneficial insects [4,5] fish [5] and other aquatic mosquito predators [6]. The use of chemicals for malaria vector control may lead to high mortalities of predators in aquatic larval habitats and a subsequent increase in mosquito larval habitat productivity [6]. There is renewed interest in establishing sustainable alternative control methods to complement existing vector control tools using biological resources. Naturally occurring predators have been shown to be a significant ecological factor in the regulation of *An. gambiae *larvae population [7-10]. Blaustein and Chase [7] found that predator and larvae associations are likely to reduce the mosquito populations and thus could be an effective management tool for their control. Predators such as notonectids [11], belostomatids [11], dytiscid beetles [8,11], crustaceans [12], copepods [13], Odonata [14,15], wolf spiders (Araneae: Lycosidae) [16] and amphibians [11] have been shown to be potential biological control agents against mosquito species in various habitats such as agricultural drainages, rice fields and small water bodies. These habitats are some of the predominant larval habitats in the region for *An. gambiae *[17]. The concept of importing non-indigenous species for biological control has come under discussion because of the potential undesirable effects of predation, parasitism, and competition on non-target native fauna and flora [18,19]. However, among the predators described above, notonectids, belostomatids, odonates, *Gambusia affins *and tadpoles have been found to coexist in natural mosquito breeding habitats in the western Kenya highlands and other parts of the world [7,16]. These predators have wide range of prey [9,20], and they are likely to regulate the abundance of larval mosquitoes that share the same habitats [17]. Predator-larvae interactions have been found to be one of the most important factors in the mortality of mosquito larvae in natural habitats [6].

The present study aimed to evaluate the predation rate and efficacy of five main mosquito larvae predators found in natural habitats against *An. gambiae *larvae in different habitat types in highlands of western Kenya. Backswimmers (Hemiptera: Notonectidae), tadpoles (Anura: Myobatrachidae), belostomatids (Hemiptera: Belostomatidae), dragon fly nymphs (Odonata: Anisoptera) and *Gambusia affins *were evaluated in both 24 hour evaluation and semi-field experiments. Evaluation of larval predator efficiency has implications for developing and establishing biological control programs against *An. gambiae *and other pest mosquito larvae.

## Materials and methods

### Study area description

24 hour evaluations (0.16256N; 34.74408E) and semi-field (0.16825N; 34.71632E) experiments were conducted in the western Kenyan highlands. Western Kenya is considered to be highly prone to malaria epidemics. Swamp reclamation for agriculture and deforestation for timber and firewood in this area have increased the density of potential breeding habitats [21]. *An. gambiae s.s *and *An. funestus *are the primary and secondary malaria vectors in this study area, respectively [17].

### Predator collection

Predator collections were made from natural mosquito breeding habitats in Iguhu village in the western Kenya highlands. Predators were sampled using a standard dipper (350 ml), transferred to basins and transported to the field insectary in Iguhu. They were subsequently placed in individual basins to avoid possible cannibalism as was found to occur in previous study [22]. Predators were introduced in habitats after a 12 hour starvation period for both 24 hour evaluations and semi-field experiments.

### Preliminary predation assessment

Preliminary experiments were set-up to ascertain which larval instar was most highly to be preyed upon by each predator. Larvae were introduced in semi-natural habitats at 7:00 Hours and counted after 12 and 24 hours. Each larval instar was tested with each predator species. The potential aquatic predators species found not feeding on any larval stage such as water beetles (Coleoptera: Hydrophilidae) were not considered for semi-field evaluation.

### 24 hour evaluation and Semi-field experimental designs

**Figure 1:**
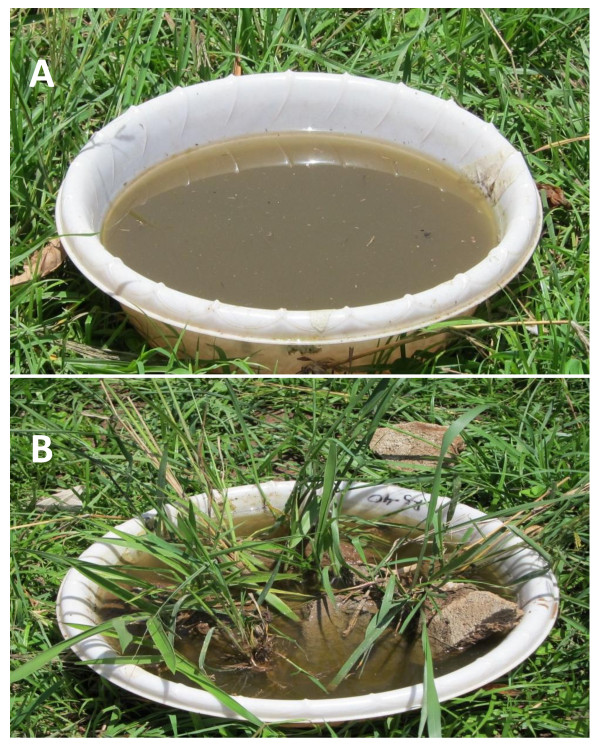
**Habitats used for 24 hour evaluation and semi-field experimental settings: habitat without (A) and with refugia (B)**.

These experiments were conducted in two habitat types, with and without refugia, in semi-natural environments. Semi-natural habitats without refugia consisted of two kilograms of soil, 2500 mls of rain water and mosquito larvae (Figure 1A). The habitats with refugia were made up of contents similar to habitats without refugia except that they contained stones and grasses which mimicked hiding structures found in natural habitats for larvae against predators (Figure 1B). The third instar larval was used in the 24 hour evaluation and semi-field experiments. Larval densities of 20, 30 and 40 larvae per basin were used to test the effects of larval density on predator efficiency.

24 hour evaluations were started in either the morning (08:00 h) or evening (18:00 h). The numbers of larvae surviving in each habitat over 24 hours were recorded. Each experiment in each density and habitat type was replicated 10 times.

In the semi-field evaluations (controlled experiments set up in field sites where natural habitats are found), experiments were set-up at 08:00 h and larvae were counted once daily until they were all either consumed or had developed to the pupal stage. Experiments with each predator for each larval density and habitat type were replicated 10 times.

### Predator midgut analysis

To verify predation of larvae, ten predators of each species were randomly selected for dissection of the midguts after the experiment. Predators were preserved in 96% ethanol immediately after being taken from experimental basins to stop further digestion of larval DNA [23]. DNA was extracted from the midgut of ten individuals of each of the five predator species tested, and a PCR reaction was performed with *An. gambiae *s.s. specific primers as described by Scott *et al*. [24]. Five μl of DNA extract was amplified in a 25 μl PCR-mix containing 1X taq Buffer (Qiagen, Valencia CA, U.S.A.), 2 mM of MgCl_2_, 0.2 mM of each dNTP, 0.5 ng/μl of primer UN [5'-GTG TGC CCC TTC CTC GAT GT-3'], 0.25 ng/μl of primer GA [5'-CTG GTT TGG TCG GCA CGT TT-3'], 0.73 ng/μl of primer AR [5'-AAG TGT CCT TCT CCA TCC TA-3'], 1 ng/μl primer QD [5'-CAG ACC AAG ATG GTT AGT AT-3'], 0.5 ng/μl primer ME [5'-TGA CCA ACC CAC TCC CTT GA-3'] and 0.05 U/μl HotstartTaq polymerase (Qiagen, Valencia CA, U.S.A.). The PCR reaction was carried out with an initial step of 10 min at 94°C followed by 30 cycles, each consisting of 5 min denaturation at 94°C, 30 s annealing at 50°C and 30 s extension at 72°C; the final cycle products were extended for 10 min at 72°C. Fragments were run through an ethidium bromide 2% agarose gel and photographed under ultraviolet light illumination.

### Data Analyses

Data analyses were done using PWAS statistics program, version 18, (SPSS Inc., Chicago) for windows and statistica version 6.0. The predation efficiency is defined as mortality rate of larval tested. Daily larval survival among predator species in 24 hour evaluation experiments was compared using chi-square tests, and predation efficiency between predator species and other factors were compared using multivariate analysis of variance (MANOVA) using the Tukey-Kramer HSD test. The comparisons of the larval survival proportions in 12 hours and 24 hours experiments (i.e. morning and evening set-ups) were compared using chi-squared with the adjusted proportion of surviving larvae. In the semi-field experiments, the influence of predator species, habitat type and prey density on larvae survivals were analyzed using MANOVA and differences were compared using the Tukey-Kramer HSD test. The daily survival rates comparisons were computed by the use of one way analysis of variance (ANOVA). The Tukey-Kramer HSD test was used for the analysis of predator species contribution towards developmental time reduction for *An. gambiae *larvae.

## Results

### Preliminary predation assessment

Preliminary predation assessments confirmed five of the eleven predators evaluated to be feeding on larvae of *An. gambiae *Notably, all of the confirmed predators demonstrated highest predation efficiency feeding on third instar larvae. Third instars were used in the subsequent 24 hour evaluation and semi-field experiments.

### 24 hour evaluation of predation efficiency

**Figure 2:**
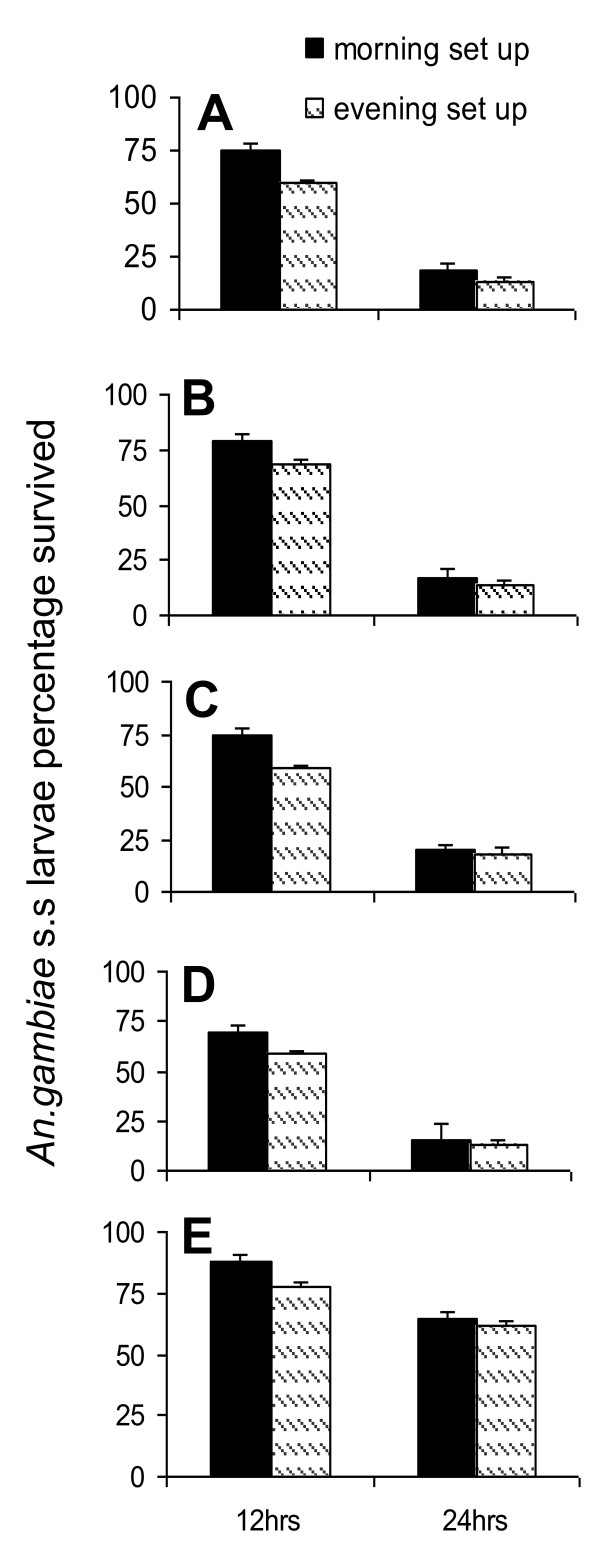
**Larval survival rate (measure of predation efficiency) after 12 Hours and 24 Hours of exposure to predators in 24 hour evaluation for morning and evening experimental settings**. From top to bottom: A) Backswimmer, B) Belestoma, C) Dragon Fly nymph, D) *Gambusia affins*, E) Tadpole.

The predation efficiency of the five predator species varied significantly in both morning (χ^2 ^= 33.06; d.f. = 12, *P *< 0.001) and evening (χ^2 ^= 40.54; d.f. = 12, *P *< 0.0001) experimental set-ups. More larvae were consumed during the night phase hours (Figure 2). Multifactorial analysis of variance (MANOVA) showed that predator species, habitat type and prey density were significant factors affecting in predation efficiency of evaluated predators (Table 1). The chi-squared test for 12 and 24 hour predation differences found no significant differences for each predator in adjusted larval survival proportions (Figure 2).

**Table 1:** The analysis of effect of combined factors in predation efficiency of aquatic predators against third instar larvae of *An. gambiae *s.s in 24 hour evaluation settings

Source of variations	F-test	p-value
Habitat types (H)	30.8 _5, 266_	< 0.0001
Prey density (P)	35.5 _10, 532_	< 0.0001
Predator species (Ps)	15.5 _20, 883_	< 0.0001
H × P	3.2 _10, 532_	< 0.0001
H × Ps	7.2 _20, 883_	< 0.0001
P × Ps	6.0 _40,1162_	< 0.0001
H × P × Ps	3.3 _40,1162_	< 0.0001

### Semi-field experiments

In the semi-field experiments, there were significant variations among the five predators relative to control in *An. gambiae *s.s. larvae daily survival rate (Table 2) and pupation rate (Table 3) reduction relative to controls (Pair-wise comparison using Tukey-Kramer HSD quartile value q* = 2.87, *P *< 0.05).

**Table 2:** The efficiency of predators in reducing the survival rates of against third instar larvae of *An. gambiae *s.s in semi-field experimental settings

Predator species	Mean (± SD)	Relative reduction (%)	Levelx
Control	0.92 ± 0.21	0	a
Tadpole	0.86 ± 0.26	6.08	a
Belestoma	0.56 ± 0.31	39.24	b
Dragonfly Nymph	0.37 ± 0.35	59.60	c
*Gambusia affins*	0.28 ± 0.32	69.69	c
Backswimmer	0.24 ± 0.33	74.30	c

Percentage of relative reduction was calculated against control population.

^x ^Levels not connected by same letter are significantly different

**Table 3:** The efficiency of predators in reducing the pupation rates in semi-field experimental settings.

Predator species	Mean (± SD)	Relative reduction (%)	Levelx
Control	99.43 ± 1.71	0	a
Tadpole	97.01 ± 3.97	2.42	a
Belestoma	56.86 ± 26.05	42.81	b
Dragonfly Nymph	19.40 ± 10.44	80.49	c
*Gambusia affins*	8.75 ± 9.49	91.20	d
Backswimmer	3.38 ± 4.14	96.61	d

Percentage of relative reduction was calculated against control population.

^x ^Levels not connected by same letter are significantly different

The variation of factors influencing pupation rates using MANOVA results showed that predator species, prey densities, habitat type, interactions between predator species and prey density, predator species and habitat type had significant influence on pupation rate (Table 4). The prey density × habitat type × predator species and the prey density × habitat type interactions had no significant influence on pupation rates (Table 4). Daily survival rates (predation rates), habitat type and predator species had a significant influence on survival rate reduction (Table 5). The prey density interactions between predator species and prey density, between predator species and habitat type, between prey density and habitat type and among predator, and between prey density and habitat type had no significant influence on the predation rates (Table 5).

**Table 4:** The analysis of effect of combined factors in pupation rate reduction efficiency of aquatic predators against third instar larvae of *An. gambiae *in semi-field settings

Source of variations	F-test	P-value
Habitat type(H)	74.61_1,324_	< 0.0001
Prey density(P)	29.59_2,324_	< 0.0001
Predator species (Ps)	1942.67_5,324_	< 0.0001
H × P	0.33_2, 324_	0.716
H × Ps	11.81_5,324_	< 0.0001
P × Ps	39.63_10, 324_	< 0.0001
H × P × Ps	0.74 _10, 324_	0.69

**Table 5:** The analysis of combined factors effects on daily survival rates (predation rates) reduction efficiency of aquatic predators against *An. gambiae *third instar larvae in semi-field settings

Source of variations	F-test	p-value
Habitat type (H)	57.154_5, 324_	< 0.0001
Prey density (P)	0.270_2, 324_	0.76
Predator species (Ps)	12.028_1, 324_	< 0.001
H × P	0.772_5, 324_	0.57
H × Ps	0.069_2, 324_	0.93
P × Ps	0.796_10, 324_	0.63
H × P × Ps	0.100_10, 324_	0.99

### Midgut analyses

**Figure 3:**
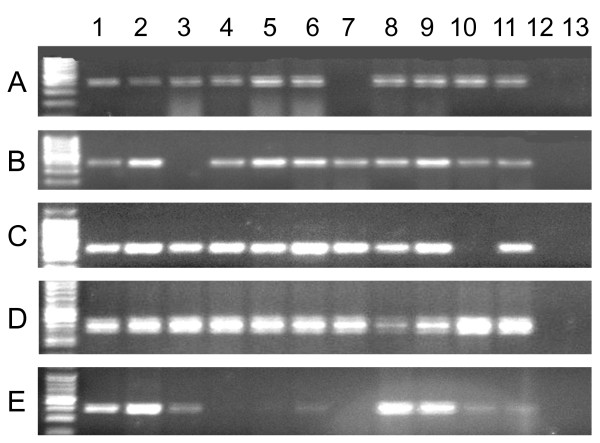
**Amplification of *An. gambiae *s.s DNA from the midguts of five predators: **A) Backswimmer, B) Belestoma, C) Dragon Fly nymph, D) *Gambusia affins*, E) Tadpole. Column: 1 = *An. gambiae *s.s. DNA, positive control; 2-11 = DNA from midguts of 10 predators; 12 = negative control, 13 = *An. arabiensis *DNA, control for primer specificity.

In the semi-field experiments, *An. gambiae *DNA was found in all five of the predators evaluated, confirming actual ingestion of larvae by each species. Of the ten replicates for each predator tadpoles, backswimmers, belestomatids, dragon fly nymphs and *Gambusia affins; *30%, 80%, 90%, 90% and 100% of the samples were positive for *An. gambiae *DNA, respectively (Figure 3).

## Discussion

The results of this study have demonstrated availability of potential biological resources for controlling malaria vectors in the western Kenya highlands. In the 24 hour evaluation experiments, all evaluated predators were shown to be more efficient nocturnal predators. *Gambusia affins *was most efficient while tadpoles were the least efficient predators among all. Habitat type (with and without refugia) had a significant effect on predation in both 24 hour evaluation and in semi-field experiments, which suggests that habitat refugia may be a significant factor in increased larval survival in habitats with efficient predators. In 24 hour evaluation experiments, the set-up time (i.e. morning or evening) had no effect on overall predation of each predator after 24 hours of observation. In semi-field experiments, larval density did not affect the predation rate, which suggests that predator effectiveness will not be hindered by this factor in the long term. The predation rate of predator species and the survival rates of *An. gambiae *larvae in habitats with and without refugia were similar to results found by other studies in Kenya and elsewhere [8,9,11,25]. In our experiments, all predators were shown to consume intermediate size prey (third instars larvae) at the highest rate. This may be due to a body size capture and handling trade-off. Similar findings have been reported in *Cx. quinquefasciatus and Ae. albopictus *predation experiments [26-28].

Differences in larvae body size and shapes of prey are known to influence predators capture and prey strategies [29,30]. Mosquito developmental stages produce drastic changes in body shape; from a linear first instar larva, through stages increasing in size and finally to a smaller, round pupa. The latter stage was generally the least or not at all preferred by predators, or similarly, the least vulnerable to predation. Behavioral analysis indicated slightly lower capture success and greater handling times for mosquito pupae than first to fourth instar larvae [29,30].

When the predation rates of the five predators were examined with respect to prey density, the rate of consumption varied among predator species. Daily and overall survival rates in semi-field experiments varied with predator species and not larval density and habitat type. The number of larvae consumed remained high in both habitat types when predators were introduced for evaluation. This reflects the combined effects of searching ability and consumption of *An. gambiae *larvae by the predator species on a temporal and spatial scale [31]. Of the predators evaluated, *Gambusia affins *and backswimmers were most efficient in predation. The predators evaluated are known to feed on other mosquito species and they have been reported to coexist in several aquatic habitat types that are readily found in the study region [10,11,32]. The effects of these aquatic predators on daily survivorship and pupation rates of *An. gambiae *s.s larvae in western Kenya are being reported here for the first time. In recent years, culicine and *Aedes *larval population regulation by dytiscid beetles have been noted in different parts of the world [33], and this study has demonstrated their efficacy in predation of *An. gambiae *larvae.

The efficacy of existing malaria vector control methods in several parts of Africa has been reduced due to insecticide resistance selection pressure among insecticide classes used for indoor residual spray and bed net treatments [34,35]. This has led to a revival of interest in the use of locally available biological resources for sustainable, cost effective control of aquatic mosquito stages. Previous studies in India [8], Kenya [36] and Australia [37] have shown the use of aquatic predators to be effective in reducing malaria vector populations and disease incidence. The integration of aquatic predators in broad scale malaria vector control campaigns may lead to more effective control programs [10,38-40].

Several studies have demonstrated strong top-down regulation of mosquito larvae by aquatic predators [41]. In the current study, variation in predation rate was mostly associated with the presence or absence of refugia and predator species. Predator candidates for the biocontrol of *An. gambiae *larvae would ideally be able to increase their population size in the absence of *An. gambiae *by relying on alternative prey [40,42]. Given that larval *An. gambiae *habitats in western Kenya often contain a suite of controphic species, future studies should address competitive advantages of co-occurring species. The positive effect of refugia on larval survivorship also suggests that species specific ability to avoid predation likely exist. While predators clearly have the ability to regulate larval populations, *An. gambiae *are typically associated with ephemeral pools where predators may not be abundant or present at all. In these scenarios, larval populations are likely regulated by hydroperiod [43] and/or controphic and intraspecific exploitative competition [7]. In habitats with larval predators, top-down and bottom-up processes are likely important as a joint determinants of community structure.

The larval and predator species composition and abundance in natural habitats are influenced by the ecological characteristics of habitat [44,45]. From the viewpoint of biological control, the aquatic predators should have a wide range of adaptability in the habitats apart from the predation of target mosquito larvae. Therefore, it may be beneficial to advocate the use of these predators in the wider community as a biological control tool against the aquatic stages of *An. gambiae *in western Kenya.

## Conclusion

Four of five predators evaluated in the 24 hour evaluation and semi-field experiments were able to consume a significant number of mosquito larvae and reduce survival and pupation rates considerably in both habitats, with and without refugia. Our results suggest that, the efficiency of a predator depends on detectability of prey in habitats. The predation risk was shown to be body size (larval instar) dependent. The evaluated predators may play an important role in larval population regulation and thereby impart a positive effect on malaria vector reduction in western Kenya.

## Competing interests

The authors declare that they have no competing interests.

## Authors' contributions

EJK conceived, designed and performed experiments. GY and AKG coordinated and supervised the study progress. EJK and ZG did data analysis and interpretation. EJK wrote this manuscript. GY, TMG, ZG, AKG, YA and MN edited the manuscript. All authors have read the manuscript and approved for submission.
